# Adaptation of motor unit synergies in the synergetic ankle plantarflexors in ambulatory persons with incomplete spinal cord injury

**DOI:** 10.1186/s12984-026-01874-2

**Published:** 2026-01-13

**Authors:** Zhihao Duan, Asta Kizyte, Emelie Butler Forslund, Elena M. Gutierrez-Farewik, Pawel Herman, Ruoli Wang

**Affiliations:** 1 Department of Engineering Mechanics, KTH MoveAbility, KTH Royal Institute of Technology, 100 44 Stockholm, Sweden; 2 Department of Neurobiology, Care Science and Society, Karolinska Institutet, 141 83 Stockholm, Sweden; 3 Aleris Rehab Station R&D Unit, 169 89 Solna, Sweden; 4 Department of Women’s and Children’s Health, Karolinska Institutet, 171 77 Stockholm, Sweden; 5 Division of Computational Science and Technology, Electrical Engineering and Computer Science, KTH Royal Institute of Technology and Digital Future, 100 44 Stockholm, Sweden

**Keywords:** HD-EMG decomposition, Muscle synergies, Coherence analysis, Factor analysis, Motor unit clustering

## Abstract

**Background:**

Spinal cord injury (SCI) often results in impaired motor control and coordination. Previous studies have highlighted the role of muscle synergies in coordinating motor tasks and their alterations following SCI. However, the adaptation in muscle synergy patterns at the motor unit (MU) level after SCI remains unexplored. This study aimed to investigate MU synergies and clustering in the synergetic soleus and gastrocnemius medialis (GM) muscles and to explore how these patterns are altered in persons with SCI.

**Methods:**

High-density electromyography (HD-EMG) was used to record MU activity in the soleus and GM muscles of fifteen participants with incomplete SCI and ten non-disabled participants during 20% and 50% maximal voluntary isometric contraction tasks. The HD-EMG signals were decomposed into individual MU spike trains. Inter-muscle coherence analysis was employed to evaluate the shared neural drive between the soleus and GM muscles, and factor analysis was performed to identify synergistic clusters of MUs innervating each muscle.

**Results:**

The results showed that both participant groups demonstrated high coherence between the soleus and GM muscles, highlighting a shared neural drive for coordinated function. However, participants with SCI showed altered coherence in the delta frequency band, with significantly higher coherence observed at 50% maximal voluntary contraction (*p* = 0.047). Additionally, factor analysis revealed that participants with SCI had a reduced proportion of MUs in the shared cluster within the GM muscle at 20% maximal voluntary contraction (*p* < 0.01).

**Conclusions:**

These findings suggested that SCI may disrupt MU synergies and clustering, potentially impairing motor coordination. This research offered valuable insights into the underlying mechanism of muscle synergies and the neural adaptations following SCI, providing crucial information for the development of future rehabilitation strategies.

**Supplementary Information:**

The online version contains supplementary material available at 10.1186/s12984-026-01874-2.

## Introduction

Neural control of movement is a highly intricate process that relies on the coordinated activity of motor units (MUs) to generate precise muscle forces as needed. Each MU, composed of a motor neuron (MN) and the muscle fibers it innervates, is activated by synaptic inputs to generate forces necessary for smooth and accurate movement. MNs receive two types of synaptic input: common input, shared across the MN pool, and independent input, unique to each MN [1, 2]. Common input is critical for regulating muscle force by coordinating the activation of a population of MNs [3], while independent input, arising from neuron-specific synaptic fluctuations such as membrane noise, is filtered by the MN pool and results in variability in spike timing [4, 5]. Traditional muscle synergies refer to coordinated activation patterns among groups of muscles, typically inferred from surface electromyographic (EMG), and are thought to be driven by a higher-level neural command that simplifies motor control [6–8]. In contrast, MU-level synergies quantify the common synaptic inputs shared among MNs, providing a more direct and physiologically grounded measure of shared neural drive. Understanding how MU-level synergies are formed and modulated is essential for elucidating the mechanisms underlying effective and adaptive movements. For instance, following spinal cord injury (SCI), these shared synaptic inputs can be disrupted, resulting in altered MU coordination, weakened or uncoordinated muscle activation, and significant difficulty in executing precise movements [9–11].

EMG signals, which capture the electrical activity of muscles, are widely used as a non-invasive method to assess the modularity of neural control and muscle synergies in persons with motor impairments. The muscle-based EMG synergy approach extracts synergy patterns based on muscle activation that represent the coordinated action of multiple muscles during specific motor tasks. Studies used EMG to assess muscle synergies during overground walking have reported that individuals with SCI exhibit a reduced number of muscle synergies compared to control groups, indicating that muscle coordination is significantly impaired following SCI [12–14]. While EMG-based synergy analysis provides valuable insights into muscle coordination patterns, it does not capture the changes in common synaptic input that drives muscle activation or the neural mechanisms underlying MU coordination after SCI. A more direct approach, analyzing MU coherence and clustering of MU spike trains, can provide an in-depth understanding of the shared synaptic input that drives muscle activation and changes in muscle synergies after SCI [1, 15–17].

The developments of high-density EMG (HD-EMG) and advanced decomposition algorithms, such as convolutive blind source separation [18] or FastICA [19], have enabled the in vivo analysis of individual MU spiking activity, offering new perspectives on assessing muscle activation at the MU level. Traditionally, the common synaptic input to an MN pool, which consists of a group of MNs that innervate the same muscle, was considered as a shared synaptic drive coordinating the entire pool. However, later studies have shown that MUs innervating the same muscle may not necessarily receive the same synaptic inputs, while MUs from different muscles can share common inputs [2, 17]. Functionally, MUs could be organized into clusters based on shared common inputs they received, forming distinct MU modes that represent coordinated MU activation patterns. These modes constitute MU synergies that coordinate activation across multiple muscles to produce specific movements. Del Vecchio et al. found that the shared synaptic inputs between two distinct MN pools in the thenar and first dorsal interosseous muscles were associated with force control [20]. Similarly, four MU synergies were found to account for over 70% of the MU activity across fourteen intrinsic and extrinsic hand muscles [21].

Techniques such as factor analysis [22], principle component analysis [23], and graph-based approaches [17] have been used to quantify and classify common synaptic inputs received by MUs across muscles into functional clusters. Hug et al. investigated correlation networks of MUs from six lower limb muscles during a multi-joint isometric task using hierarchical clustering. They found that MUs were grouped based on their shared common inputs, therefore supporting the concept that movements are governed by a small number of MU clusters receiving shared common inputs [17]. Similarly, Nuccio et al. used factor analysis to classify MUs in agonistic knee extensors (vastus lateralis and vastus medialis muscles) into three clusters in individuals with anterior cruciate ligament reconstruction. They observed that, on the surgically repaired side, a higher proportion of MUs exhibited discharge behavior more strongly associated with the synergistic muscle than with their innervated muscle, compared to the unaffected limb [24]. These studies demonstrated that the functional clusters of MUs and their shared neural input might be affected by the trauma. However, the extent to which these synergies are disrupted following neurological disorders, i.e., SCI, remains unclear. Addressing this knowledge gap is essential, as MU synergies represent fundamental neural strategies in muscle force modulation and movement coordination.

While previous studies have primarily investigated muscle synergies from a global perspective (across multiple joints), the organization of synergies within functionally related muscles, and their alteration in neurological disorders remains poorly understood. This understanding is critical for elucidating the mechanisms underlying joint-level force modulation and coordination. Therefore, this study aims to investigate MU synergies and clustering between two major ankle plantarflexors, soleus (SOL) and gastrocnemius (GM) muscles, and to evaluate potential differences between ambulatory persons with incomplete SCI and controls at different levels of submaximal contraction. We hypothesize that individuals with incomplete SCI will exhibit altered neural drive coherence between the SOL and GM muscles compared to able-bodied subjects. Furthermore, we expect altered MU clustering patterns in individuals with incomplete SCI.

## Methods

### Participants

The dataset included fifteen participants with sub-acute and chronic SCI (ten males; age 54.9 ± 13.6 years; height 173.7 ± 8.1 cm; weight 78.9 ± 18.8 kg) and ten non-disabled participants (six males; age 50.4 ± 12.6 years; height 174.8 ± 8.7 cm; weight 74.2 ± 14.2 kg). The SCI group consisted of a subset of participants who were initially recruited for a study conducted by Butler Forslund et al. [25]. The inclusion criteria were designed to ensure participants exhibit less than full plantarflexor strength while retaining sufficient capacity to complete the test procedures. All participants were classified as paraplegia with incomplete SCI, categorized as grade D according to the American Spinal Injury Association scale, indicating that they had full arm function and reduced function in trunk and legs. The median time since the injury was 6.2 years (interquartile range: 9.6 years). The injury levels of the participants with SCI are as follows: six participants in the thoracic region (T1-T6), four in the thoracic region (T7-T12), four in the lumbar region, and one in the cervical region (with no motor deficit at cervical level). All participants with SCI underwent clinical evaluation and demonstrated less than full but adequate muscle strength to complete the test procedures. All participants in the control group had no known neurological disorders or lower limb injuries within the six months prior to the study. Ethical approval for the study was obtained from the Swedish Ethical Review Authority (2020–02311, 2020–07067, and 2022-00629-02). All participants gave informed written consent. Data collection was conducted at the Promobilia MoveAbility Lab at KTH Royal Institute of Technology (Stockholm, Sweden).

### Experimental protocol and data collection

Participants were seated comfortably in a chair with the ankle joint positioned at 0° and the knee flexed at 90°, while one leg was securely attached to a stationary isometric dynamometer (OT Bioelettronica, Turin, Italy, sampling frequency 100 Hz). In the SCI group, for participants with asymmetrical motor function, measurements were taken on the side with weaker muscles, as identified by an experienced physiotherapist. For those with symmetrical motor function, the side with the most reduced sensory function was measured. In the control group, the side for measurement was randomly selected. HD-EMG signals were recorded by a 32-channel grid (GR10MM0804, OT Bioelettronica) placed on the SOL, and a 64-channel grid (GR10MM0808, OT Bioelettronica) on the GM, following the recommended placement guidelines by SENIAM [26]. Maximal isometric voluntary contraction of plantarflexors was determined as the peak value obtained from two repeat trials of maximal voluntary contraction (MVC), each lasting 3 s, with a one-minute rest period. Participants were then instructed to perform four repetitions of submaximal isometric plantarflexion contractions at either 20% or 50% of their MVC in a randomized order. Each contraction followed a trapezoidal torque profile (a 5-second ramp-up, a 4-second plateau, and a 5-second ramp-down) guided by a visual cue, with a 10-second rest period between each repetition. The torque was measured using a dynamometer equipped with an S-beam bidirectional load cell and a single-channel general-purpose amplifier (Forza, OT Bioelettronica). Simultaneously, HD-EMG signals were recorded in monopolar mode, band-pass filtered between 10 Hz and 500 Hz, and sampled at 2048 Hz using a multichannel acquisition system (Quattrocento, OT Bioelettronica).

**Fig. 1:**
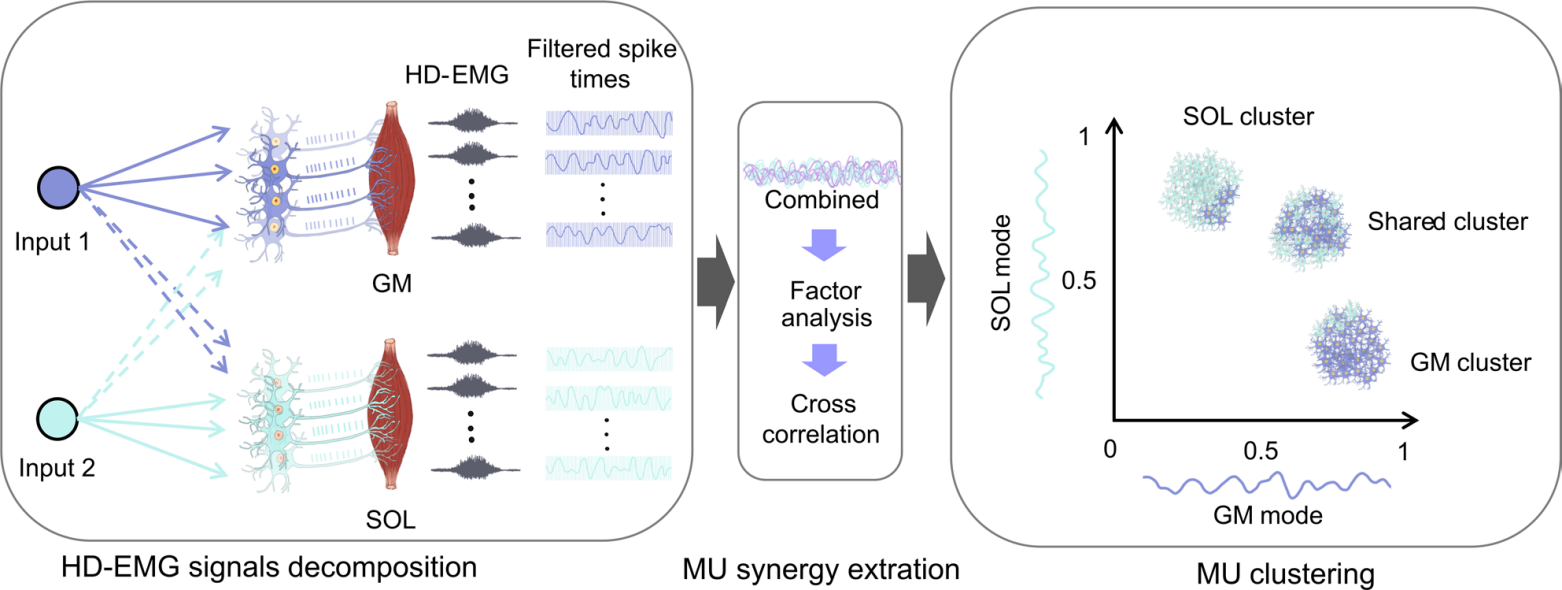
The detailed workflow of the study. HD-EMG signals recorded from the SOL and GM muscles were decomposed into MU spike trains. These decomposed spike trains were then filtered using a 400-ms Hanning window. The filtered spike trains were combined and factor analysis was applied to identify two primary modes (SOL and GM mode). Correlations between each filtered spike train and each mode were clustered based on their correlation with these modes

In this study, the schematic workflow of data processing consisted of several key steps (Fig. 1): (1) HD-EMG signals decomposition (2) MU synergy extraction, and (3) MU clustering.

### HD-EMG decomposition

The HD-EMG signals were filtered by a second-order Butterworth filter with a bandwidth of 20 Hz to 500 Hz. Subsequently, the signals were decomposed into individual MU spike trains using the convolutive blind source separation method via DEMUSE software (version 6.0; University of Maribor, Slovenia) [18, 27], with 50 iterations specified for the decomposition process. In brief, the EMG signals were first extended and whitened, and then a fixed-point algorithm was employed to maximize sparsity to identify the MU spike trains embedded within the signals. The decomposed spike trains were then subjected to visual review and manual editing to ensure accuracy, following the guidelines provided in the manual [28]. For further analysis, only MU spike trains meeting the following criteria were included: a pulse-to-noise ratio > 30 dB [29] and the trial that each muscle has at least three decomposed MUs.

### Inter-muscle coherence

To estimate the shared neural input between MUs between SOL and GM muscles, coherence was calculated between the cumulative spike trains (CST) of MUs from each muscle [30]. The magnitude-squared coherence was computed between the CSTs of MUs from each muscle using Welch’s averaged periodogram method, with non-overlapping one-second Hanning windows. Each CST was defined as the summed discharge times of two randomly selected MUs from the same muscle. For each participant, coherence between the CSTs of the SOL and GM muscles was calculated over 100 iterations. In each iteration, two MUs were randomly selected from each muscle to form the CST. When the number of identified MUs is small, coherence is calculated by resampling with replacement to reach the desired iterations, ensuring sufficient data for reliable estimates. The final coherence value for each participant was derived as the average coherence across these iterations. To account for variability in the spiking onset and duration of each MU, the analysis focused on a specific time window of approximately 5 s, which included the plateau phase of the contraction and parts of the ramp-up and ramp-down phases. The window was selected by balancing the inclusion of active MUs and the duration of their spiking activity, aiming to maximize the number of MU while retaining sufficient signal length for analysis. Only MUs exhibiting continuous firing, with no pauses exceeding 0.5 s during the selected time window, were included in the analysis. The areas under the coherence curve, referred to as integrated coherence, were calculated within three frequency bands: delta (0–5 Hz), alpha (6–12 Hz), and beta (15–30 Hz) bands. To establish a baseline, the average coherence in the frequency range of 250–500 Hz where no coherence was expected [30, 31], was computed and subtracted from the coherence profile.

### Factor analysis

The factor analysis was performed following the procedures detailed in Del Vecchio et al. [22]. The spike trains of each MU were filtered by convolving with a 400-ms Hanning window. To maintain consistency, factor analysis was performed using the same time intervals as the coherence analysis. The filtered MU spike trains from the GM and SOL muscles were then pooled together. Two main muscle modes, the SOL mode and GM mode, were identified through factor analysis of the pooled filtered MU spike trains. The two primary factors extracted can account for the majority of the variance in the dataset, enabling the characterization of distinct muscle activation patterns [22]. The percentage of variance explained by the two extracted factors was calculated and reported in the Supplementary Material (Fig. 1). The identification was performed using the *factoran* function in MATLAB R2022b. Correlations between the filtered spike trains of each MU and identified muscle modes were subsequently calculated. Based on their correlations with muscle modes, the pooled MUs were classified into three clusters: the GM cluster, SOL cluster, and shared cluster. The classification criteria were as follows: MUs were assigned to the GM cluster if their correlation with the GM mode was at least 50% greater than their correlation with the SOL mode. Similarly, MUs were assigned to the SOL cluster if their correlation with SOL mode was at least 50% greater than their correlation with the GM mode. MUs exhibiting similar correlations with both the SOL and GM modes were grouped into the shared cluster. The proportion of decomposed MUs in each cluster was calculated separately for each muscle.

### Computer simulation

To evaluate whether the method used in this study can successfully categorize MUs into different clusters based on the neural input they receive, a simplified leaky integrate-and-fire (LIF) model was used to simulate spiking trains of 300 MNs (Eq. 1). This simplified model included only two key parameters: soma size Ds and MN inert period IP [32]. In this simplified LIF model, the membrane resting potential was set to zero, while the membrane threshold *V*_*th*_ was set to 27 mV, consistent with typical reported values [32, 33]. The electrophysiological parameters Ds and IP were randomly sampled from those reported in our previous study [34], which employed an integrated approach combining HD-EMG decomposition and MN modelling using the LIF framework to estimate these parameters.

1 $$\begin{gathered} \tau \frac{{d{V_m}}}{{dt}}=R \cdot I(t) - {V_m}{\text{ if}}:{V_m}>{V_{th}}, \hfill \\ {\text{ }}{V_m}=0{\text{ for duration }}IP \hfill \\ \tau =\frac{{2.3 \cdot {{10}^{ - 9}}}}{{Ds_{{\text{ }}}^{{1.48}}}},R=\frac{{5.1 \cdot {{10}^{ - 5}}}}{{Ds_{{\text{ }}}^{{2.43}}}} \hfill \\ \end{gathered} $$

where *V*_*m*_ represents the membrane potential, *τ* the time constant, *I(t)* the total synaptic input, and *R* the membrane resistance.

**Fig. 2:**
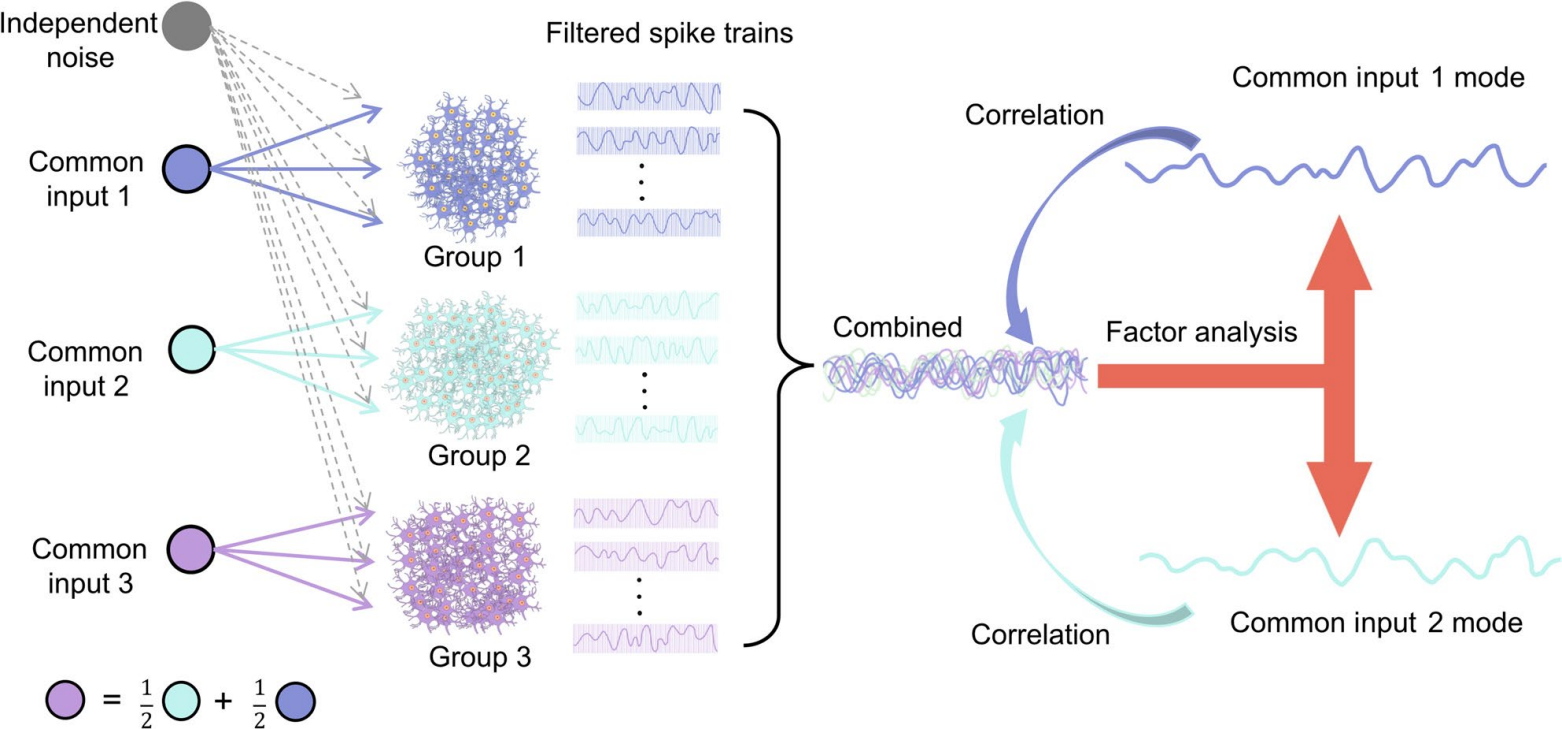
Schematic workflow of computational simulation. Three groups of MNs (each has 100 MNs) were simulated using the modified LIF mode with distinct common inputs. Two uncorrelated, orthogonal signals (common input 1 and common input 2) were generated to drive MUs in group 1 and group 2, respectively. A third input (common input 3) was created as a combination of common input 1 and 2 to drive the MNs in group 3. Each MN also received an independent input modelled as white Gaussian noise. The output spike trains were then filtered and combined, followed by factor analysis to identify two modes. The correlations between each filtered spike train and each mode were subsequently calculated to classify the MUs into different clusters

The schematic workflow of the simulation was illustrated in Fig. 2. The simulated MNs were randomly divided into three groups, each comprising 100 MNs. The total synaptic input for each MN was modeled as the linear summation of common and independent inputs, both simulated as white Gaussian noise, as expressed in Eq. (2):

2 $${I_i}(t)={C_{g(i)}}(t)+{\epsilon _i}(t)$$

where ${I_i}(t)$ is the total synaptic input to MN *i*; ${C_{g(i)}}(t)$is the common input to the group $g(i)$ that MN *i* belongs to, and ${\epsilon _i}(t)$ is the independent input specific to MN *i*. $t$denotes the time.

Three distinct common inputs were generated, one for each group of MNs. The first two common inputs were orthogonal, while the third was a composite input formed by combining the first two, with each contributing 50% to the total weight. Independent inputs were simulated separately for each MN, ensuring that each MN received a unique independent input. The simulated input duration was 7 s, with the mean of the total synaptic total input set to be 8 nA to generate physiologically realistic MU spike intervals. The variances of the common and independent inputs were equal (4 nA^2^), with a bandwidth of 0–2.5 Hz for common inputs, and a bandwidth of 0–50 Hz for independent inputs [23]. To examine the robustness of our simulation results, we also performed additional analyses using pink noise as the common synaptic input; the corresponding results are presented in the Supplementary Material.

The first second and last seconds of filtered spike trains were excluded from the analysis [22]. The remaining 5 s of data were used for further filtering and factor analysis. This duration was chosen to align with the time window used for spike trains in the experimental data, which typically lasts around 5 s. Following factor analysis, two primary modes were identified. Then, the correlations between the filtered spike trains of each MU and these modes were computed, and the MUs were subsequently classified into three clusters based on their correlations with each mode. The classification accuracy was calculated for each group of MNs. The simulation was repeated 500 times, and the average classification accuracy for each group was computed to evaluate the overall simulation performance. For each cluster, the centroid was defined as the mean position of all MUs within that cluster in the two-dimensional correlation space. The mean Euclidean distance was calculated as the average Euclidean distance of all MUs in a cluster from its centroid, providing a measure of cluster compactness.

### Statistical analysis

The primary outcome parameters for the MU coherence and clustering analysis were integrated coherence and the proportion of identified MUs assigned to each cluster. For each group, the mean integrated coherence within the delta, alpha, and beta bands and the proportion of identified MUs assigned to the three clusters for the SOL and GM muscles, were calculated by averaging the values across participants. The normality of mean integrated coherence was assessed using the Shapiro-Wilk Test. If the normality assumption was met, group differences were evaluated using the Welch two-sample t-test with a significance level of *p* < 0.05. If the normality assumption was violated, the Mann-Whitney U test was used instead. Differences in the proportion of identified MUs assigned to each cluster between the Control and SCI groups, MVC levels, and muscles were analyzed using a linear mixed-effects model in R (version 4.3.3). The model included group, MVC level, muscle, and cluster as fixed effects. The participant was treated as a random effect to account for the inter-subject variability in the number of decomposed MUs. Significance was determined at *p* < 0.05.

## Results

To investigate MU synergies and clustering in the SOL and GM muscles, we first decomposed HD-EMG signals recorded from the SOL and GM muscles during isometric plantarflexion at 20% and 50% of MVC. We then assessed the presence of shared neural input between the SOL and GM muscles through between-muscle coherence analysis of the MU spike trains. To characterize MU clustering patterns within each muscle, we applied factor analysis to the decomposed MU spike trains to classify them into distinct clusters. Finally, we conducted a computational simulation to validate the clustering method and ensure the reliability of the classification approach.

### Decomposition

In total fourteen participants with SCI and eight controls met the inclusion criterion of identifying at least three MUs per muscle at 20% MVC. At 50% MVC, eleven participants with SCI and six control participants met this criterion and were included. In the control group, an average of 11.3 ± 5.1 MUs for the SOL and 10.9 ± 9.9 MUs for the GM were decomposed at 20% MVC, while 8.5 ± 3.4 MUs for SOL and 12.2 ± 9.0 MUs for the GM at 50% MVC. In the SCI group, an average of 11.8 ± 5.1 MUs were decomposed for the SOL and 6.9 ± 2.8 MUs for the GM at 20% of MVC, while at 50% MVC, the averages were 11.3 ± 5.1 MUs for the SOL and 11.1 ± 9.9 MUs for the GM.

### Inter-muscle coherence

**Fig. 3:**
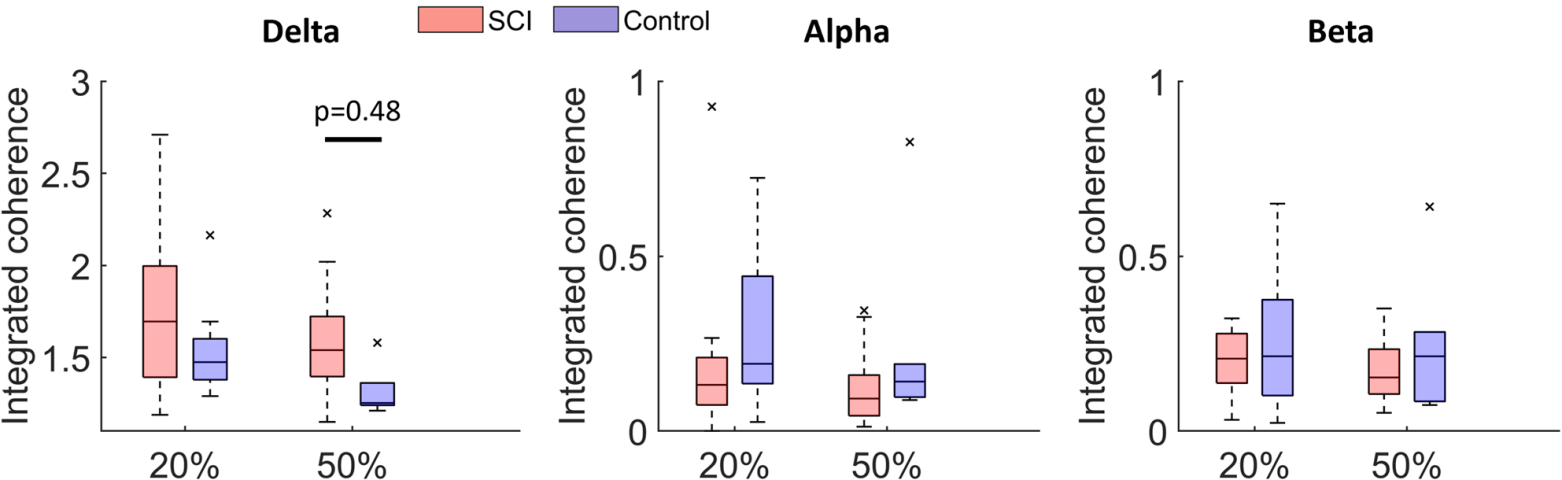
Comparison of integrated coherence across frequency bands. Integrated coherence in the delta, alpha, and beta bands was averaged for SCI and control groups at 20% MVC and 50% MVC. X-axis: contraction intensity (% MVC). Note that the y-axis scale for the delta band differs from those used for the alpha and beta bands. ^∗^indicates a significant difference between groups

Both groups show a high integrated coherence between the SOL and GM muscles, particularly within the 0–5 Hz range (Fig. 3). At 20% MVC, no significant differences can be observed between the SCI and controls in any frequency band (SCI vs. Control: delta 1.75 ± 0.41 vs. 1.55 ± 0.28; alpha 0.19 ± 0.23 vs. 0.29 ± 0.25; beta 0.32 ± 0.38 vs. 0.26 ± 0.21). At 50% MVC, the SCI group shows significantly higher integrated coherence in the delta band (SCI vs. Control: 1.60 ± 0.33 vs. 1.32 ± 0.14, *p* = 0.048). However, no significant differences are observed in the alpha or beta bands, although the SCI group shows a trend toward lower integrated coherence (SCI vs. Control: alpha 0.13 ± 0.11 vs. 0.25 ± 0.29; beta 0.18 ± 0.10 vs. 0.25 ± 0.21). Exact p-values for all comparisons are reported in Supplementary Table S1.

### Factor analysis

**Fig. 4:**
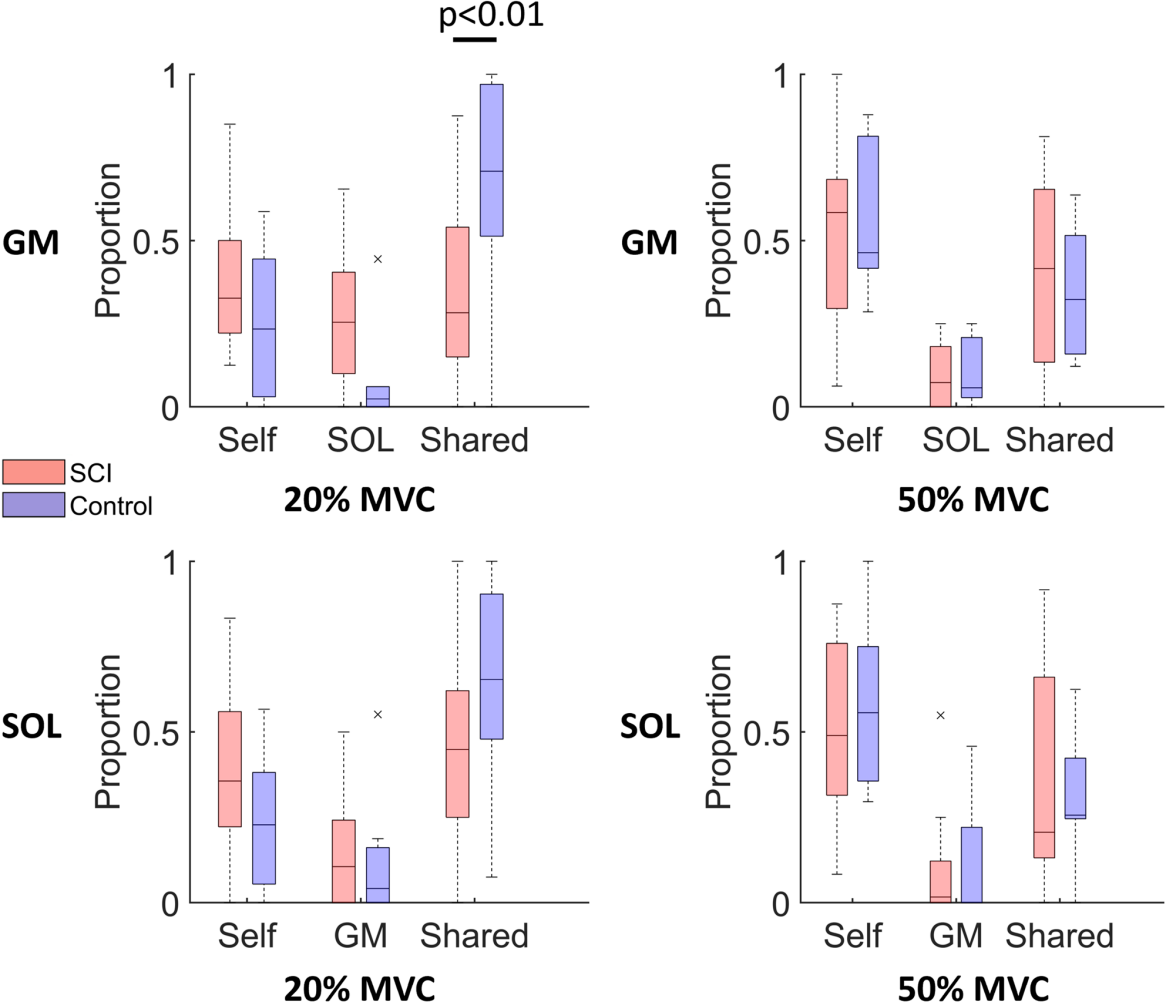
Comparison of the proportion of identified MUs assigned to different clusters. The proportion of identified MUs assigned to three clusters within the GM and SOL muscles at 20% and 50% MVC levels was averaged for SCI and control groups. Clustering was based on the correlations of MUs with each muscle mode: (1) Self cluster - MUs most strongly correlated with their respective muscle mode (2), Other (SOL or GM) cluster - MUs most strongly correlated with the mode of other muscle, and (3) Shared cluster - MUs most strongly correlated with both the GM and SOL modes. ^*^indicates a significant difference between groups. *X-axis* MU clusters

At 20% MVC, in controls, the highest proportion of identified MUs in GM are grouped within the shared cluster, followed by the self cluster, and the lowest proportion in the other (SOL cluster) (Fig. 4). In contrast, the SCI group exhibits a nearly equal distribution of the decomposed MUs among three clusters (Table 1). Compared to controls, the SCI group shows a significantly lower proportion of identified MUs assigned to the shared cluster (*p* < 0.01). In the SOL, the clustering pattern of MUs follows similar trends to those observed in the GM for both groups. No significant differences are observed in the proportion of identified MUs assigned to each cluster between the GM and SOL muscles (see Supplementary Table S2).

At 50% MVC, both the GM and SOL show similar trends in the proportion of identified MUs assigned to each cluster in both SCI and control groups, with the highest proportion of MUs assigned to the self cluster, followed by the shared cluster and the lowest proportion in the other cluster (Table 1).

**Table 1:** Comparison of the proportion of identified MUs assigned to clusters between groups for the GM and SOL muscles. P-values indicate differences in the proportion of identified MUs assigned to each cluster between groups, analyzed using a linear mixed-effects model

	Clusters	20% MVC			50% MVC		
		Control	SCI	p-value	Control	SCI	p-value
GM	Shared (%)	67.3 ± 34.6	35.5 ± 26.4	< 0.01	34.6 ± 20.0	40.5 ± 27.9	0.61
	Self (%)	25.1 ± 23.3	38.3 ± 21.0	0.22	55.4 ± 23.7	50.5 ± 28.9	0.67
	SOL (%)	7.7 ± 15.1	26.2 ± 20.5	0.08	10.0 ± 10.4	9.1 ± 10.1	0.94
SOL	Shared (%)	64.4 ± 30.7	46.8 ± 27.9	0.09	30.1 ± 20.9	39.9 ± 33.8	0.46
	Self (%)	23.7 ± 20.1	39.9 ± 24.8	0.12	58.6 ± 28.4	50.6 ± 29.0	0.55
	GM (%)	12.0 ± 18.9	13.3 ± 14.1	0.90	11.3 ± 19.1	9.5 ± 17.0	0.89

To investigate how MU clustering patterns change between 20% MVC and 50% MVC contraction levels, we compared the distribution of MUs within the same muscle across these two contraction levels. In the control group, a significantly lower proportion of identified MUs are classified in the shared cluster (*p* = 0.01), and a higher proportion in the self cluster (*p* = 0.02) at 50% MVC compared to 20% MVC in the GM. In contrast, the SCI group showed no significant changes in MU cluster distribution in the GM between 20% and 50% MVC (see Supplementary Table S3). In the SOL, the control group exhibited a significant decrease in the proportion of identified MUs classified in the shared cluster with increasing force demand (*p* = 0.01), accompanied by a significant increase in the proportion of identified MUs assigned to the self cluster (*p* < 0.01). In contrast, the SCI group showed no significant changes in MU cluster distribution across force levels.

### Computer simulation

**Fig. 5:**
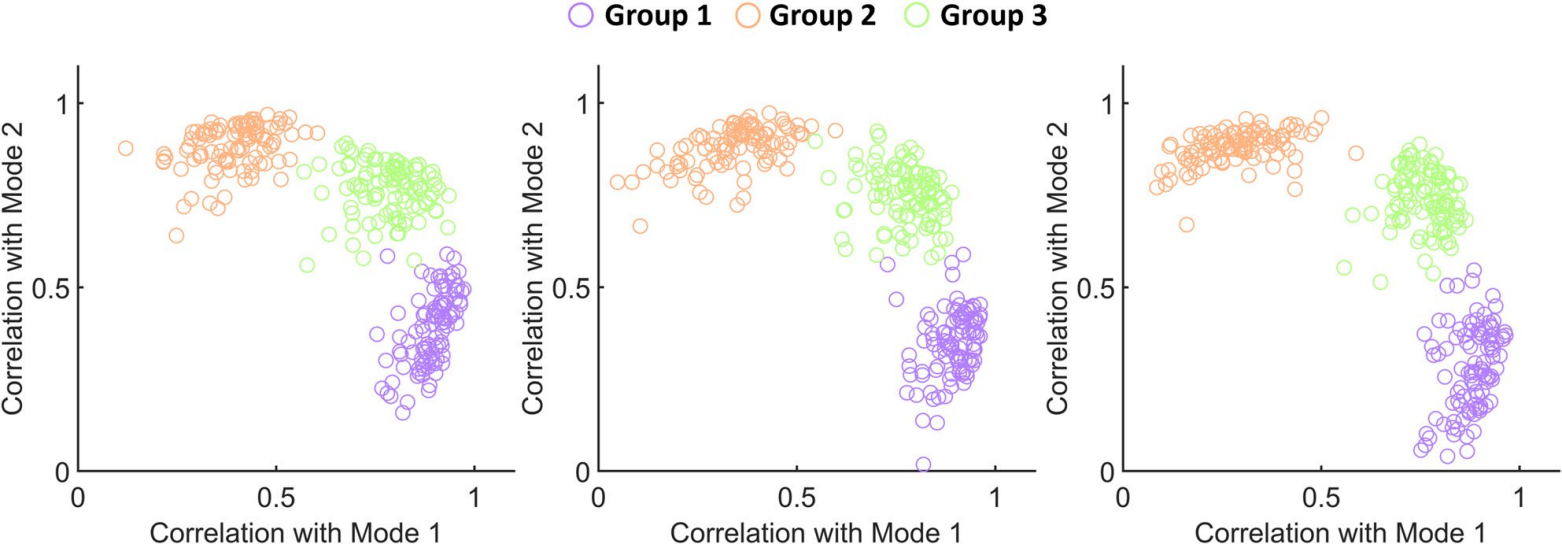
Three simulation examples illustrating MU correlations with two muscle modes from factor analysis. The plots were generated under the same input configurations. The x-axis represents the correlation coefficient of each MU with Mode 1, and the y-axis represents the correlation coefficient with Mode 2. Different colors indicate the type of common input received by each MU (100 MUs per color): purple, receiving only common input 1; orange, receiving only common input 2; and green, receiving a combination of common inputs 1 and 2 with equal weights (0.5 each)

The simulation results show that the simulated MUs can be successfully categorized into three distinct clusters according to the common input they received (Fig. 5). A high classification accuracy can be observed: 98.01 ± 5.44% for simulated MUs for group 1 (received common input 1), 98.26 ± 4.63% for group 2 (received common input 2), and 80.21 ± 14.12% for group 3 (received the combination of the common inputs). The centroid locations and mean Euclidean distances further characterize the clustering: for group 1, the centroid was at (0.85 ± 0.03, 0.20 ± 0.11) with a mean Euclidean distance of 0.10 ± 0.02; for group 2, the centroid was at (0.22 ± 0.11, 0.84 ± 0.03) with a mean distance of 0.11 ± 0.02; and for group 3, the centroid was at (0.73 ± 0.06, 0.61 ± 0.10) with a mean distance of 0.13 ± 0.02. These results demonstrate that the simulated MUs were tightly clustered around their respective centroids.

## Discussion

This study analyzed MU synergies and clustering of the synergetic ankle plantarflexors, the SOL and GM muscles to investigate potential neural strategies for muscle force modulation and coordination in ambulatory persons with incomplete SCI. The results revealed comparable intra-muscular coherence between SOL and GM muscles in both groups, with the only exception of significantly higher coherence in the delta band in the SCI group. As the contraction level increased, the control group showed a decrease in the proportion of MUs assigned to the shared cluster and a corresponding increase in the self cluster. In contrast, the SCI group showed no significant changes in MU clustering across contraction levels. In addition, no differences in the proportion of MUs assigned to clusters were found between the GM and SOL muscles in either group at the same contraction levels. However, at 20% MVC, the SCI group showed a significantly reduced proportion of MUs assigned to the shared cluster in the GM muscle. These findings, for the first time at the level of individual MU spike trains, highlight changes in MU synergies and clusters following SCI, offering valuable insights into the disruptions in muscle coordination and neural mechanisms at the MU level.

The observed MU synergies between the SOL and GM muscles confirm the presence of a shared neural drive that facilitates their coordinated function during motor tasks. Inter-muscular coherence analysis has been proven to be an effective method for assessing the connectivity between MU pools across different muscles [16, 30], enabling the quantification of the degree of synchrony in MU firing. The connectivity, often represented by the shared neural drive, is determined by the common input received by a group of MUs, resulting in synchronized MU firing [1, 2]. Overall, high coherence was observed between the SOL and GM in both SCI and control groups, particularly within the lower frequency range. Since the low-frequency components (< 5 Hz) of the neural drive are primarily translated into muscle force [35, 36], the high coherence observed in the delta band suggested a high degree of MU synchronization in the SOL and GM muscles for force generation. This synchronization likely contributes to their functional coordination and efficient movement pattern. Our observation also aligns with a previous study by Laine et al. which revealed that MUs in the synergistic thigh muscles, i.e., vastus medialis and vastus lateralis, showed higher coherence in the low-frequency range (< 5 Hz), indicating that synergistically activated muscles share a substantial proportion of their synaptic input [16].

At a moderate level of muscle contraction (50% MVC), participants with SCI showed significantly higher delta band coherence between the SOL and GM muscles, indicating enhanced low-frequency connectivity between synergetic muscles. This enhanced low-frequency synchrony may reflect a compensatory increase in shared neural drive to generate sufficient force and stabilize motor function despite impaired descending control. Previous studies using conventional EMG measurement have reported reduced muscle coordination and adaptation in muscle synergies in persons with SCI during activities such as walking, trunk stabilization, and cycling [14, 37, 38]. Thomas et al. found that weakened triceps muscles in persons after SCI were primarily due to reduced common input to triceps MNs, as evidenced by prolonged mean central nervous system conduction times to the triceps during radial nerve electrical stimulation [9]. Similarly, Bao et al. observed a reduction in synaptic inputs into the extensor digitorum communis muscle in the delta band, as revealed through coherence analysis [10]. These findings suggest that impaired descending control after SCI limits the central nervous system’s ability to coordinate MU activity across muscles. As a compensatory response, the nervous system may increase low-frequency coherence in the delta band to pool synaptic drive across MUs, thereby maintaining overall muscle coordination and stabilizing motor output. Alternatively, the same increase in low-frequency coherence may represent a maladaptive consequence of disrupted descending inputs, leading to excessive synchronization that reduces the flexibility and precision of force modulation. Computational modeling has shown that low-frequency modulation of common input can result in oscillations in muscle force within the lower frequency range [39]. These oscillations may compromise muscle force steadiness, as the periodic fluctuations in force result in less stable and more variable output [40]. Thus, altered low-frequency coherence in SCI may reflect both compensatory and maladaptive processes: supporting stability and force generation, while impairing precise force control potentially contributing to deficits in force steadiness, postural stability, and gait.

Based on our simulation using the LIF model, the factor analysis can effectively categorize MUs based on their correlation with each muscle mode, particularly in classifying MUs that received uncorrelated common inputs. However, MUs receiving mixed common inputs from both SOL and GM modes were more challenging to classify. Vecchio et al. demonstrated that the classification accuracy of the MUs receiving mixed inputs was affected by signal length, as longer signals achieved better classification accuracy [22]. In our study, the data length in the simulation was kept consistent with the recorded experimental data, thereby strengthening the reliability of our results. However, it is worth noting that the simulation used 100 MUs per group, substantially higher than the number of MUs available experimentally. This discrepancy between the simulation and experimental data may limit the applicability of the simulation findings. We classified the decomposed MUs into three clusters using a criterion requiring the MU to show at least 50% higher correlation with one mode compared to the other. Such criteria yielded the best classification accuracy in our computer simulation. However, when a MU received unequal proportions of two common inputs (e.g., 20% from common input 1 and 80% from common input 2), this approach could misclassify the MU in the cluster associated exclusively with common input 2, despite it belonging to the shared cluster. Future improvement in criteria that account for varying levels of shared input could enhance the accuracy of clustering MUs receiving mixed input proportions.

The results revealed that the distribution of MU clusters within the synergetic ankle plantarflexors at a low contraction level (20% MVC) was significantly affected in persons with SCI. Specifically, a decreased proportion of MUs assigned to the shared cluster was observed, indicating disrupted synergies between MUs in these muscles. Such disruption likely compromises muscle coordination and force steadiness, both critical for maintaining postural control and performing low-force functional tasks. Previous studies have reported that neurological disorders can negatively affect force steadiness during submaximal contractions by impairing the ability to activate multiple MUs concurrently and to maintain their asynchronous firing [11, 40, 41]. Consistently, the present findings suggest that persons with incomplete SCI may have a diminished capacity to recruit and coordinate MUs across the SOL and GM muscles, leading to impaired force steadiness and disrupted low-force muscle synergies around the ankle. The reduced proportion of shared MUs may also be attributed to MU loss following SCI, as reported by Robert et al. [42]. , which can lead to muscle denervation and impaired muscle coordination. Collectively, these alterations in MU clustering may contribute to common SCI-related motor deficits, including reduced postural stability, abnormal gait, and difficulty performing precise, low-force motor tasks. However, it is worth noting that at the 50% MVC level, no significant difference was observed in the proportion of identified MUs assigned to each cluster between the SCI and control groups. This convergence may be partly explained by the recruitment of larger, higher-threshold MUs with faster-twitch properties in both the SOL and GM muscles at higher contraction levels, resulting in more similar MU populations and thereby reducing the apparent group differences. Additionally, the lack of observed differences may also be attributed to the limited number of decomposed MUs at this higher force level due to the current decomposition method, which might not be sufficient to reveal the differences.

As the contraction level increased, the distribution of MU clusters within the SOL and GM muscles changed, characterized by a decreased proportion of MUs in the shared cluster and an increased proportion in the self cluster. Previous studies have discussed synergies in terms of both neural and task-based origins [43, 44]. By examining MU synergy within individual muscles, our clustering analysis captures patterns of common neural input that reflect the neural organization underlying muscle coordination. At the same time, the observed MU clusters could also reflect task-based synergies, since different contraction levels may recruit different MU clusters to achieve specific force outputs. Thus, while our findings highlight a strong neural contribution, we acknowledge that task-related factors may also shape the distribution of MU clusters. Physiological properties of MUs may further explain the observed changes. Schmied et al. [45]. found that higher synchronization occurred in smaller MUs with slower twitches, which were more active at lower force levels, while larger MUs with faster twitches at higher force levels [46, 47]. According to Henneman’s size principle [48], larger MUs are recruited to meet the higher force demands. We consider that these larger MUs are more likely to belong to the self cluster, which received synaptic input exclusively to the respective muscle, potentially explaining the observed cluster proportions. Interestingly, the proportion of identified MUs assigned to each cluster was similar between the SOL and GM muscles within the same group and contraction levels. This convergence likely reflects the shared anatomical and fiber-type characteristics of the two muscles. Both contain a high proportion of slow-twitch fibers and contribute to postural control and sustained plantarflexion contractions, acting synergistically to generate plantarflexion torque. This consistent trend also suggests a coordinated recruitment strategy across the SOL and GM muscles, likely driven by shared neural control mechanisms regulating MU behavior across the ankle plantarflexor muscles. The organization of MUs into distinct functional clusters supports the hypothesis that the central nervous system uses these functional clusters to reduce the dimensionality of motor control [2, 17]. The observed alterations in these functional clusters in persons with incomplete SCI imply an adaptation in the neural strategies to coordinate the simultaneous activation of the SOL and GM muscles in response to the injury.

There were several limitations in this study. First, the decomposition method used in the analysis allowed for the identification of only a small proportion of MUs. This limited sample of decomposed MUs restricted the interpretative power and ability to generalize our findings, although the number of MUs we decomposed was still comparable to those reported in the literature [49]. Additionally, the inclusion threshold of at least three decomposed MUs per muscle was set due to the difficulty of obtaining sufficient MUs from the SOL and GM muscles, as their anatomical characteristics - such as deeper location and larger size - can lead to increased overlap of action potentials, thereby making accurate decomposition and identification of individual MUs more challenging [49]. It may introduce variability in the factor analysis and affect the stability and generalizability of the results. A potential improvement would be the use of alternative decomposition methods, such as the convolution kernel compensation-peel off method [50], which has been shown to increase the number of identified MUs. Second, all identified MUs from the SOL and GM muscles were used to extract two MU modes, without accounting for the potential imbalance in MU number between muscles. The number of MUs included from each muscle may influence the resulting synergy structure; therefore, future studies with a sufficient number of identified MUs could control for this by using an equal number of MUs from each muscle when calculating MU synergies. Third, our SCI cohort was heterogeneous in both injury level and time since injury. Variation in corticospinal tract integrity associated with injury level may differentially influence the distribution of common synaptic input to MNs, thereby affecting MU synergies. Higher-level lesions (e.g., cervical) may cause greater loss of descending drive than lower thoracic injuries, resulting in distinct MU coordination patterns. These effects can be further influenced by time since injury and rehabilitation history. Such variability may increase inter-subject differences and obscure group-level effects. Another limitation was the small sample size, particularly at the 50% MVC contraction level, where fewer control participants met the inclusion criteria. This constrains interpretation, as non-significant results may reflect either a true physiological finding or limited statistical power. Significant findings should also be interpreted cautiously due to the small sample size. Future studies with larger sample sizes are needed to strengthen the robustness and generalizability of these findings and to enable subgroup analyses that clarify how specific injury characteristics influence MU-level synergies. In the SCI group, only the more impaired limb was assessed to capture injury-related changes, which may have amplified differences with controls and limited generalizability. Comparing both limbs in the future would provide a more complete picture of MU adaptations following incomplete SCI.

In conclusion, the results revealed altered coherence between MUs in the SOL and GM muscles post in complete SCI, particularly in the delta band, indicating a higher shared neural drive. Furthermore, the distribution of MUs across functional clusters at lower contraction levels in the SCI group was significantly altered, with a reduced proportion of shared MUs suggesting disrupted MU synergies and coordination. As contraction intensity increased, the proportion of MU clusters shifted with a decreased proportion of the shared cluster and an increased proportion of the self cluster within each muscle. However, the proportion of MU clusters remained similar between the SOL and GM muscles despite the increase in contraction intensity, reflected a coordinated recruitment strategy between synergistic muscles. These findings emphasized the impact of incomplete SCI on muscle coordination and highlighted the potential of MU synergies and clustering methods for investigating neurological adaptations and potential to inform future advanced rehabilitation strategies.

## Supplementary Information

Below is the link to the electronic supplementary material.

Supplementary Material 1

## Data Availability

Datasets are available upon reasonable request to the corresponding author.

## Abbreviations

CST: Cumulative spike trains
EMG: Electromyographic
GM: Gastrocnemius
HD-EMG: High-density electromyographic
LIF: Leaky integrate-and-fire
MN: Motor neuron
MU: Motor unit
MVC: Maximal voluntary contraction
SCI: Spinal cord injury
SOL: Soleus
