# Supplementary material for: Adaptation of motor unit synergies in the synergetic ankle plantarflexors in ambulatory persons with incomplete spinal cord injury
Source: J Neuroeng Rehabil. 2026 Jan 13;23:42. doi: 10.1186/s12984-026-01874-2 (PMC12849599; doi:10.1186/s12984-026-01874-2)
Supplement: Supplementary file 1 — Supplementary Material 1 [file 12984_2026_1874_MOESM1_ESM.pdf]

Variance explained by extracted factors in the experimental data

To evaluate the appropriateness of extracting two factors in the experimental data, we examined the percentage of variance explained by successive factors (Fig. 1). At 20% MVC, the first two factors accounted for 79.9 ± 9.7% of the variance in the SCI group and 87.5 ± 6.2% in controls. At 50% MVC, they explained 86.7 ± 4.6% and 84.1 ± 5.1% in the SCI group and control group, respectively. These values are consistent with previous reports of MU-level factor analyses of lower limb muscles, which typically show that two components capture the majority of the variance in MU discharge rates [1-3].

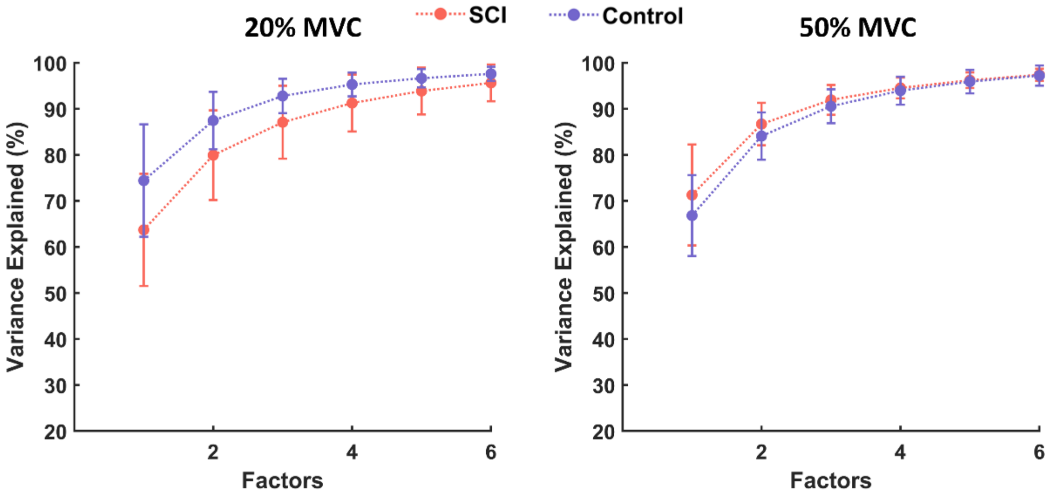

Figure 1. Percentage of variance explained by the extracted factors in the experimental data for each group and contraction level. Variance explained was calculated using factor analysis applied to all filtered MU spike trains decomposed from the soleus (SOL) and gastrocnemius medialis (GM) muscles. Data are presented as mean ± SD across participants. Results are shown separately for the SCI group (red) and control group (blue) during isometric plantarflexion at 20% MVC (left panel) and 50% MVC (right panel).

[1] Del Vecchio A, Marconi Germer C, Kinfe TM, Nuccio S, Hug F, Eskofier B, et al. The Forces Generated by Agonist Muscles during Isometric Contractions Arise from Motor Unit Synergies. J Neurosci. 2023;43(16):2860-73.

[2] Rossato J, Avrillon S, Tucker K, Farina D, Hug F. The Volitional Control of Individual Motor Units Is Constrained within Low-Dimensional Neural Manifolds by Common Inputs. J Neurosci. 2024;44(34).

[3] Cabral HV, Inglis JG, Pourreza E, Dos Santos MA, Cosentino C, O'Reilly D, et al. A single low-dimensional neural component of spinal motor neuron activity explains force generation across repetitive isometric tasks. iScience. 2025:113483.

Table S1. P-values for the comparison of integrated coherence between the soleus (SOL) and gastrocnemius medialis (GM) muscles. The table reports p-values for the comparisons between the SCI and control groups at 20% and 50% maximal voluntary contraction (MVC) across three frequency bands: delta (0–5 Hz), alpha (6–12 Hz), and beta (15–30 Hz).

MVC	Band	p-value
20%	Delta	0.211
	Alpha	0.330
	Beta	0.973
50%	Delta	0.047
	Alpha	0.216
	Beta	0.317

Table S2. Comparison of MU clusters between GM and SOL. P-values for comparison of MU cluster proportion differences in each cluster between the GM and SOL muscles for each group at same contraction levels using the linear mixed-effects model.

p-values	20% MVC		50% MVC	
	Control (GM vs SOL)	SCI (GM vs SOL)	Control (GM vs SOL)	SCI (GM vs SOL)
Share	0.81	0.19	0.72	0.96
Self	0.91	0.85	0.79	0.99
Others	0.73	0.14	0.92	0.97

Table S3. Comparison of MU clusters between 20% and 50% MVCs. P-values represent the statistical significance of differences in the proportions of MUs within each cluster between 20% and 50% MVC, analyzed separately for each group within the GM and SOL muscles using a linear mixed-effects model.

p-values	GM (20% vs 50% MVC)		SOL (20% vs 50% MVC)	
	Control	SCI	Control	SCI
Share	0.01	0.60	0.01	0.50
Self	0.02	0.20	<0.01	0.29
Others	0.85	0.07	0.96	0.70

Supplementary methods: pink noise simulation

To examine the robustness of the simulation model, we repeated the analysis using pink noise instead of white Gaussian noise to model the common synaptic input. Pink noise was chosen because motor outputs generated by the CNS often exhibit fractal-like properties, in contrast to Gaussian distributions, potentially providing a more physiologically plausible representation of synaptic drive. All other simulation parameters were kept identical to those described in the main manuscript.

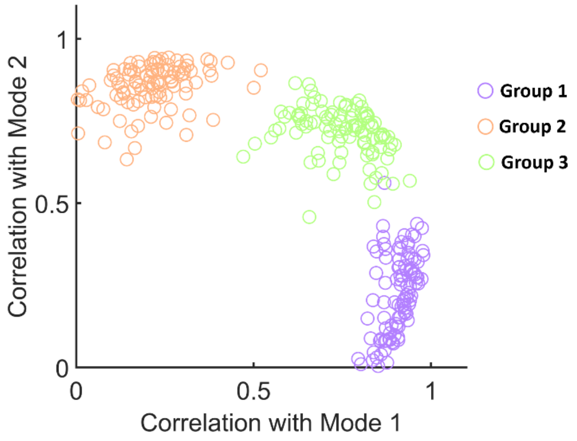

Figure 2. An example of simulation results using pink noise, showing correlations between each MU and two muscle modes. The x-axis represents correlation with Mode 1, and the y-axis represents correlation with Mode 2. Different colors indicate the type of common input received by each MU, with 100 MUs per color: purple MUs receive only common input 1, orange MUs receive only common input 2, and green MUs receive a combination of common inputs 1 and 2 with equal weights (0.5 each).

The simulation results show that the simulated MUs can be successfully categorized into three distinct clusters according to the common input they received (Fig 2). A high classification accuracy can be observed: 97.83 ± 6.82% for simulated MUs for group 1 (received common input 1), 97.21 ± 8.27% for group 2 (received common input 2), and 81.80 ± 13.91% for group 3 (received the combination of the common inputs). The centroid locations and mean Euclidean distances for each cluster were as follows: group 1, centroid at (0.88 ± 0.03, 0.22 ± 0.13) with a mean distance of 0.09 ± 0.02; group 2, centroid at (0.22 ± 0.14, 0.88 ± 0.04) with a mean distance of 0.10 ± 0.02; and group 3, centroid at (0.75 ± 0.08, 0.62 ± 0.13) with a mean distance of 0.12 ± 0.02. Notably, these results are

comparable to those obtained using Gaussian noise, confirming that the clustering results are robust and not dependent on the assumption of Gaussian noise for the common synaptic input.
